# The significance of right ear auditory processing to balance

**DOI:** 10.1038/s41598-022-24020-z

**Published:** 2022-11-17

**Authors:** Hanna Putter-Katz, Niza Horev, Erez Yaakobi, Ella Been

**Affiliations:** 1 Department of Communication Sciences and Disorders, Faculty of Health Professions, Ono Academic College, Zahal 104, Kiryat Ono, Israel; 2 Speech and Hearing Unit, ENT Department, Tel Aviv Sourasky Medical Center, Tel Aviv, Israel; 3 Faculty of Business Administration, Ono Academic College, Kiryat Ono, Israel; 4 Department of Sports Therapy, Faculty of Health Professions, Ono Academic College, Kiryat Ono, Israel

**Keywords:** Neuroscience, Physiology, Risk factors

## Abstract

Although the association between balance and hearing thresholds at different frequencies in the right/left ear is crucial, it has received scant empirical attention. Balance is widely ignored when evaluating hearing in adults. This study examined the relative contribution of left versus right ear hearing at different frequencies to balance, and the mediating role of suprathreshold speech perception on age-balance associations. Pure tone hearing thresholds (500–4000 Hz), suprathreshold speech perception, balance, and risk of falling were evaluated in 295 adults. The results indicate that the right ear contributes more to balance than the left ear. This might imply dominance of the left hemisphere in processing hearing cues for balance. Frequencies within the speech range (500/1000/2000 Hz) were correlated with balance and mediated the interaction between age and balance. These results should be considered when tailoring hearing and balance rehabilitation programs.

## Introduction

Beginning with the discovery of the left-hemispheric dominance of language^1–3^ there has been a consensus that practically all higher functions, including memory, learning, perception, spatial cognition, attention, complex motor skills, and emotion processing show some degree of lateralization^4–6^. Specifically, right ear processing is significantly more efficient for speech stimuli^7^. In recent years, a growing body of evidence has suggested that hearing cues contribute to balance^8,9^. Studies show that auditory information can be integrated with vestibular, somatosensory, and visual signals to improve balance, orientation, and gait^10–13^. Despite its importance, to the best of our knowledge, the relative contribution of the right/left ear to balance has never been explored.

Shayman et al.^12^ reported that external auditory input contributes meaningful information to vestibular self-motion cues in a frequency-dependent manner. They showed that auditory cues significantly improve sensitivity to self-motion perception below 0.5 Hz, whereas vestibular cues contribute more at higher frequencies. However, the ways in which hearing thresholds at different frequencies potentially influence balance control remain unclear.

To improve the ecological validity of the human hearing-balance relationship Criter & Gustavson^14^ and Carpenter & Campos^15^ recommended that future research should use real life environments and functional indices rather than relying solely on a laboratory-based approach consisting of pure-tone hearing thresholds. One of the first signs of hearing deterioration is difficulty in understanding speech in challenging everyday listening situations^15–19^. However, very little is known about the interaction between the deterioration of speech perception and balance.

Falling and its consequences have a significant impact on individuals (loss of quality of life, nursing home admissions) and society (healthcare costs)^9,13^. Early detection of balance disorders and possible interventions can potentially reduce falling and prevent its consequences^13^. Recent studies have shown that auditory information can be integrated with vestibular, somatosensory, and visual signals to improve balance, orientation, and gait^10–13^. However, hearing status is rarely taken into account when evaluating gait and balance^8,9^.

To respond to these needs, the current study examined the interaction between hearing and balance in a group of adults, using functional indices of hearing and balance. It then explored the relative contribution of the left versus right ear at different frequencies to balance. The findings should lead to a better understanding of the age-balance association.

## Results

The descriptive statistics and inter-correlations for hearing and balance measures are presented in Tables 1 and 2, respectively. Table 3 provides a list of abbreviations.

**Table 1:** Means (M) and standard deviations (SD) for the hearing and balance variables.

Measures	Right ear		Left ear	
	M	SD	M	SD
WIN 50% SNR	4.65	3.23	4.94	3.79
PTA 1 (in dB)	19.81	8.20	20.33	9.69
PTA 2 (in dB)	20.75	9.72	21.68	11.00
4000 Hz	25.02	14.75	27.34	15.48
2000 Hz	17.83	10.39	18.44	12.24
1000 Hz	19.41	9.06	19.08	10.48
500 Hz	21.97	9.20	21.68	9.78
TUG (seconds)	8.77	1.44		

WIN, words in noise, SNR, signal to noise ratio in dB, PTA, pure tone average in dB, TUG, timed up and go (in seconds).

**Table 2:** Inter-correlations (Pearson correlations) between the age, hearing, and balance variables.

	Age	RE WIN 50% SNR	LE WIN 50% SNR	RE PTA 1	LE PTA 1	RE PTA 2	LE PTA 2	RE 4000 Hz	RE 2000 Hz	RE 1000 Hz	RE 500 Hz	LE 4000 Hz	LE 2000 Hz	LE 1000 Hz	LE 500 Hz
Age															
RE WIN 50% SNR	.26***														
LE WIN 50% SNR	.24***	.53***													
RE PTA 1	.27***	.32***	.35***												
LE PTA 1	.27***	.31***	.37***	.79***											
RE PTA 2	.35***	.40***	.44***	.85***	.75***										
LE PTA 2	.32***	.40***	.44***	.72**	.91***	.81***									
RE 4000 Hz	.36***	.38***	.42***	.53***	.53***	.88***	.69***								
RE 2000 Hz	.31***	.39***	.45***	.83***	.71***	.91***	.75***	.69***							
RE 1000 Hz	.20***	.23***	.23***	.92***	.73***	.75***	.62***	.40***	.66***						
RE 500 Hz	.21***	.18**	.19***	.83***	.60***	.52***	.44***	.24***	.44***	.76***					
LE 4000 Hz	.35***	.37***	.41***	.47***	.63***	.70***	.86***	.75***	.58***	.36***	.23***				
LE 2000 Hz	.28***	.41***	.45***	.68***	.86***	.77***	.91***	.62***	.79***	.57***	.36***	.69***			
LE 1000 Hz	.19***	.24***	.28***	.75***	.91***	.61***	.79***	.34***	.59***	.73***	.60***	.44***	.70***		
LE 500 Hz	.17**	.23***	.24***	.74***	.83***	.54***	.63***	.28***	.47***	.71***	.75***	.36***	.54***	.80***	
TUG	.16**	.21***	.15**	.22**	.17**	.16**	.14*	.06	.17**	.23***	.18***	.06	.15**	.17**	.19***

RE, right ear, LE, left ear, WIN, words in noise, PTA, pure tone average, TUG, timed up and go. *p < .05, **p < .01, ***p < .001.

**Table 3:** Abbreviations.

Abbreviation	Full name
PTA	Pure tone average
WIN	Words in Noise test
HWIN	Hebrew version of the Words in Noise test
TUG	Timed Up and Go test
Hz	Hertz
dB	Decibel
SNR	Signal- to- noise ratio

Balance was significantly correlated with the Pure Tone Average 1 (PTA1, calculated as the average hearing threshold at 500 Hz, 1000 Hz, and 2000 Hz), Pure Tone Average 2 (PTA2, calculated as the average hearing threshold at 1000 Hz, 2000 Hz, and 4000 Hz), the Words In Noise test WIN 50% SNR, and with hearing thresholds at 500 Hz, 1000 Hz, and 2000 Hz but not at 4000 Hz, in both ears. Age was significantly correlated with the PTA 1/2, WIN 50% SNR, hearing thresholds (500 Hz, 1000 Hz, 2000 Hz, 4000 Hz) and balance in both ears.

### Mediation effects

**Figure 1:**
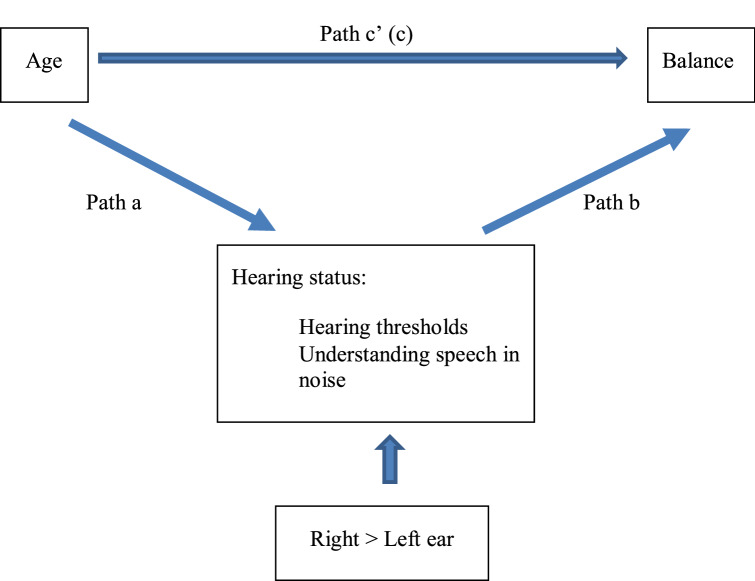
The mediating effects of hearing status on age and balance. Directionality was determined based on research demonstrating the impact of hearing and hearing loss on balance^8,14,15^. c = Total effect. C’ = the effect of age on balance, controlling for the effect of hearing status.

Since there was a significant correlation between age and balance, age and hearing, and balance and hearing, the mechanism underlying the observed relationship between age and balance (Fig. 1) was explored further. To test for a mediation effect, we used the PROCESS add-on in SPSS. This macro calculates two regression analyses. The first estimates the effect of age on hearing measures (path a). The second regression estimates the effect of hearing measures on balance (path b) controlling for age. The cross-product a*b is considered an estimation of the indirect effect of age on balance via hearing measures. The significance of the indirect effect was calculated with a 95% confidence interval bootstrapping approach because the sampling distribution of the indirect effect is known to be skewed. Cases where the 95%CI does not include zero are equivalent to a significant effect at alpha < 0.05.

Significant associations were observed between age and hearing measures (Path a: supplementary data are available online Table S1-7). For the right ear, WIN 50% SNR, PTA1 and hearing thresholds (500 Hz, 1000 Hz, 2000 Hz) were associated with balance after controlling for individuals’ age (Path b). PTA2 and hearing thresholds 4000 Hz were not associated with balance after controlling for individuals’ age (supplementary data are available online Table S1-S7). By contrast, for the left ear, only PTA1, 500 Hz, and 1000 Hz were associated with balance after controlling for age. The WIN 50% SNR, PTA2, 2000 Hz, and 4000 Hz were not associated with balance (Path b: supplementary data are available online Table S1-S7).

The results for the indirect analyses (Path a*b) are presented in Table 4.

**Table 4:** Regression results for the simple mediation of the right and left ears on the association between age and balance through hearing measures (Path a*b).

	Right ear				Left ear			
	β	SE	LLCI 95%	ULCI 95%	β	SE	LLCI 95%	ULCI 95%
**Indirect effects and significance using the normal distribution**								
**Bootstrap results for indirect effects**								
WIN 50% SNR	.07	.03	**.018**	**.128**	.04	.03	−.001	.101
PTA1	.05	.02	**.017**	**.094**	.04	.02	**.002**	**.076**
PTA2	.04	.02	−.006	.090	.03	.02	−.006	.080
500HZ	.03	.02	**.007**	**.069**	.03	.02	**.005**	**.061**
1000HZ	.04	.02	**.012**	**.075**	.03	.01	**.004**	**.060**
2000HZ	.04	.02	**.003**	**.088**	.03	.02	−.002	.075
4000HZ	.003	.02	−.044	.050	.003	.02	−.041	.047

Unstandardized regression coefficients are reported. Bootstrap sample size = 5000. LL, lower limit; CI, confidence interval; UL, upper limit. Significant mediation effects are in bold**.** As shown, five mediation effects were observed for the right ear but only three for the left ear. The significance of the indirect effect was calculated with a 95% confidence interval bootstrapping approach because the sampling distribution of the indirect effect is known to be skewed. Cases where the 95%CI does not include zero are equivalent to a significant effect at alpha < .05.

**Figure 2:**
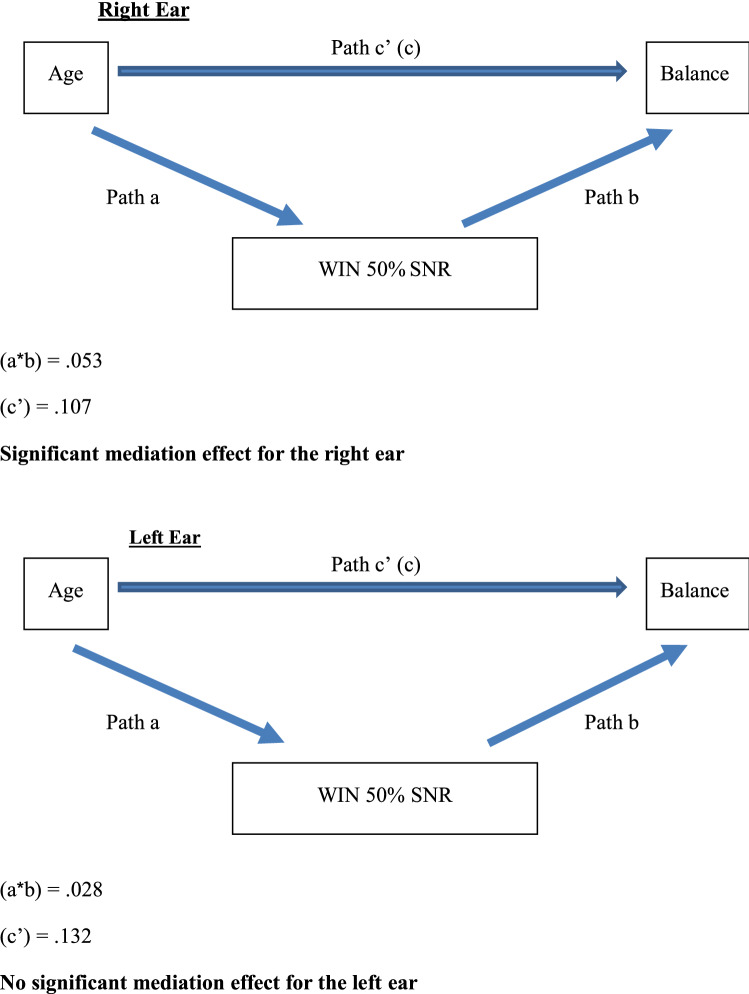
The mediating effects of WIN 50% SNR on age and balance for the right and left ears.

As shown in Table 4, five mediation effects were observed for the right ear but only three for the left ear. For the right ear, WIN 50% SNR, PTA1 and hearing thresholds (500 Hz, 1000 Hz, 2000 Hz) fully mediated the association between age and balance. Bootstrap results showed that the bootstrapped 95% CI around the indirect effect did not include zero. On the other hand, hearing thresholds of 4000 Hz and PTA2 did not mediate the association between age and balance (Fig. 2).

For the left ear, PTA1, 500 Hz and 1000 Hz mediated the association between age and balance. Bootstrap results for these measures showed that the bootstrapped 95% CI around the indirect effect did not include zero (Path a*b). On the other hand, WIN 50% SNR, PTA2 and hearing thresholds of 2000 Hz, and 4000 Hz did not mediate the association between age and balance. Bootstrap results for these measures showed that the bootstrapped 95% CI around the indirect effect included zero.

To determine whether the right or left ear was more likely to mediate the association between age and balance, we conducted a parallel mediation model in which both ears competed with each other as an explanatory mechanism. As presented in Table 5, for WIN 50% SNR and PTA1, the right ear emerged as a significant mediator whereas the left ear was not significant. For PTA2, neither ear was more dominant.

**Table 5:** Comparing indirect effects for the right vs. left ears.

	Right ear				Left ear			
	β	SE	LLCI 95%	ULCI 95%	β	SE	LLCI 95%	ULCI 95%
**Indirect effects and significance using the normal distribution**								
**Bootstrap results for indirect effects**								
WIN 50% SNR	.010	.005	**.001**	**.002**	.002	.004	−.006	.012
PTA1	.014	.007	**.002**	**.029**	−.002	.006	−.013	.010
PTA2	.008	.008	−.010	.025	.002	.007	−.011	.018

Unstandardized regression coefficients are reported. Bootstrap sample size = 5000. LL, lower limit; CI, confidence interval; UL, upper limit. Significant mediation effects are in bold**.** The significance of the indirect effect was calculated with a 95% confidence interval bootstrapping approach because the sampling distribution of the indirect effect is known to be skewed. Cases where the 95%CI does not include zero are equivalent to a significant effect at alpha < .05.

## Discussion

The results of the current study indicate a stronger contribution of the right ear to balance than the left ear. Consistently, the correlations between the right ear and balance were higher than those for the left ear. In the right ear, almost all the hearing measures mediated the relationship between age and balance (WIN 50% SNR, PTA 1, hearing thresholds 500 Hz/1000 Hz/2000 Hz). By contrast, in the left ear, only PTA 1 and hearing thresholds of 500 Hz/1000 Hz mediated this interaction. Hearing measures for the right ear evidenced a stronger mediation effect than the left ear with respect to the interaction between age and balance (Tables 4, 5). These results may point to the dominance of the left hemisphere in processing hearing cues for balance.

To the best of our knowledge, this is the first study to suggest hemispheric lateralization and left hemisphere dominancy to account for the hearing-balance relationship. This should come as no surprise since all the major cognitive functions including language, spatial and emotional processing are lateralized^1–6^. The right ear advantage is well-known for the processing of verbal stimuli, reflecting left hemispheric dominance for language^4–6^. Studies have argued for the enhanced role of the left hemisphere in the control of motor actions^20^. Although hemispheric function for postural control and balance is not fully understood, most studies indicate that the right cerebral hemisphere plays a more prominent role in the efferent processes responsible for balance control^21–24^. For example, Golomer et al.^21^ found that right hemispheric visual dominance is particularly useful for postural control in complex equilibrium conditions. On the other hand, Cioncoloni et al.^25^ suggested that the left hemisphere plays a critical role in the selection of the appropriate postural control strategy. These findings emphasize the fact that the cerebral role in postural control and the cortical mechanisms of spatial hearing are complex processes, and more research is needed to elucidate them^25,26^.

Very little is known about the contribution of hearing at different frequencies to balance^12^. The current findings suggest that frequencies within the speech range (500/1000/2000 Hz) are correlated with balance. Both PTA 1 (the average of the hearing thresholds of 500 Hz, 1000 Hz, and 2000 Hz) which is the clinical predictor of the speech reception threshold (SRT), and WIN (speech perception in noise) in the right ear mediated the interaction between age and balance. These results raise the possibility that deterioration of speech perception in the presence of noise might indicate balance deterioration. However, pure tone thresholds of 4000 Hz, in both ears, were not correlated with balance and did not mediate the relationship between age and balance. Since age-related hearing loss is characterized by bilateral hearing loss above 2000 Hz, this strengthens the claim that the relationship between hearing and balance is affected by factors other than age-related hearing loss.

The current study shows that hearing interacts significantly with balance in adults (Table 2). This is consistent with data reported in Agmon et al.^8^, Criter & Gustavson^14^, Carpenter & Campos^15^, Li et al.^27^, Rumalla et al.^28^, Campos et al.^29^ and Doettl et al.^30^. Specifically, Lin and Ferrucci^10^ found that for every 10 dB increase in hearing loss, the probability of an individual reporting a fall increased by 1.4. The interaction between hearing and balance has also been reported in patients with hearing loss^14,31^. Impaired balance was also found to exist in younger populations with hearing impairments^32,33^. This association between hearing loss and falls may be accounted for by several mechanisms: (a) physiological mechanisms that may influence auditory and postural systems. These could involve a concomitant dysfunction of both cochlear and vestibular sensory organs given their shared location within the labyrinth in the inner ear, or age-related changes in the corpus callosum that could affect both hearing and walking^8,34^; (b) cognitive mechanisms such as paying attention to postural control might tap cognitive resources. Fewer cognitive resources and less attention due to hearing loss may impair postural balance in real life situations and increase the risk of falling^8,35,36^; (c) behavioral mechanisms such as hearing loss might influence spatial orientation, social parameters, and the interaction between the effects of reduced mobility and reduced auditory inputs. Hearing deterioration may thus restrict a person’s ability to monitor and perceive auditory environmental cues that provide spatial orientation^8^.

Consistent with previous studies^37,38^ the current study found a decline in hearing and balance with advancing age. Furthermore, the findings indicated that balance was correlated with hearing, even when controlling for age. As demonstrated in the current study, hearing mediated the interaction between age and balance. This implies that one of the reasons for the deterioration of balance with advancing age may result from hearing deterioration. This finding is supported by previous studies indicating that balance deterioration is positively correlated with the extent of hearing deterioration in hearing-impaired populations^33,39,40^.

The current study used hearing tests that simulate everyday hearing situations (WIN), in addition to the commonly used index of hearing thresholds (pure tone thresholds in the range of 0.5–4.0 kHz). These tests were selected based on recommendations in previous studies^14,15,26,41^, in an attempt to better preserve the ecological validity of the human hearing-balance relationship. It is also important to note that since balance is a very complex function, the results of the balance test used in the current study (TUG) might have been affected by other factors such as peripheral hearing, vestibular, and visual factors. However, TUG is considered to be a good diagnostic tool for balance and risk of falling^42^, and is often being used in research evaluating balance in adult populations^14,43^. Further research should explore these topics in a variety of populations in different age groups and while using a variety of hearing/balance measures and pathologies.

Thus overall, the correlations between hearing and balance and the mediating effect of speech range frequencies on the age and balance relationship suggest that difficulties in understanding speech in adults over the age of 45y may indicate reduced balance and might imply the need for a balance evaluation. At the same time, balance difficulties may indicate the need for a hearing evaluation. Thus, the current study supports previous research recommending the evaluation of balance in individuals with hearing deterioration^9,44^, in order to potentially reduce falling and prevent its consequences. The relatively greater contribution of the right ear to balance, compared to the left ear, should be considered during hearing evaluation and rehabilitation. Hearing may thus contribute to balance in addition to visual, vestibular, and proprioceptive input.

## Materials and methods

### Participants

A sample of 295 community dwelling adults (181 female and 114 male) aged 46–75 years (58.5 ± 6.1), participated in this study. Written informed consent was obtained from all subjects. All participants underwent two hearing tests (Standard Pure-Tone Audiometry test and Words-in-Noise—WIN), one balance test (Timed Up and Go—TUG) and the Montreal Cognitive Assessment—MoCA. All methods were performed in accordance with the relevant guidelines and regulations.

Exclusion criteria included poor physical health^38^, mobility using walking aids, and suspected presence of mild cognitive impairment as defined by the MoCA < 26/30^45^. After signing the informed consent form and completing the MoCA questionnaire, the participants were administered the hearing and the balance tests.

### Hearing and balance evaluation

Hearing in the right and left ears was evaluated using Standard Pure-Tone Audiometry, and the Hebrew version of Words-in-Noise (HWIN) test^46,47^. To assess hearing thresholds, the Standard Pure-Tone Audiometry^48^ was administered at octave levels from 500 to 4000 Hz using a HARP mobile audiometer with TDH-50 earphones (Grason-Stadler Inc, Eden Prairie, MN; Guymark UK Limited, West Midlands, UK). The pure tone average 1 (PTA1) was calculated as the average hearing threshold at 500 Hz, 1000 Hz, and 2000 Hz. PTA1 is regarded as a predictor of the speech reception threshold. The pure tone average 2 (PTA2) was calculated as the average hearing threshold at 1000 Hz, 2000 Hz, and 4000 Hz. PTA2 emphasizes the weight of high frequencies to hearing.

The WIN is a word-recognition test to assess speech perception in noise^46^. The Hebrew version of the WIN consists of two lists of 35 common consonant–vowel-consonant (CVC) words mixed with 6 talkers’ babble at 7 signal- to- noise ratios (SNRs) from 24 to 0 dB_SNR_ in 4 dB increments. The two lists were presented to each subject, one for each ear for open set identification^47^. The total number of correctly identified words and the 50% point in dB_SNR_ (WIN 50% SNR) for each ear was calculated using the Spearman-Karber Eq. ^49^.

Performance-based balance was measured using the timed up and go test (TUG). The TUG is a widely used instrument that examines balance, functional mobility, and risk of falling across multiple adult populations^14,50–53^. The test requires the subject to stand up, walk 3 m, turn, walk back, and sit down. Time taken to complete the test is strongly correlated with level of balance and functional mobility. Cognition was assessed by the Hebrew version^54^ of the MoCA^45^.

### Statistical analysis

The statistical analysis was performed using IBM SPSS Statistics for Windows v.24. The data were expressed as the mean ± standard deviation (SD). Pearson’s correlation analysis was used to determine the correlation between age, hearing tests and balance tests. A value of p < 0.05 was considered statistically significant. To examine the mediational role of the hearing measures, the PROCESS macro^41^ Model 4 was used to calculate four sets of regressions (Fig. 1). The first set of regressions examined the associations between the predictors (age) and mediating variables (hearing measures), Path a. The second set of regressions examined the links from the mediators (hearing measures) to the outcomes (balance) controlling for age, Path b. The third set of regressions examined the direct associations between the predictors (age) and the outcome (balance), Path c. The fourth set of regression examined the direct associations between the predictors (age) and the outcome (balance) controlling for the mediators (hearing measures), Path c'. To test the significance of the indirect effects of age on balance through hearing deterioration, the bootstrapping approach was used and the 95% CI for the indirect effects on 5,000 resamples was calculated^55^.

### Ethical considerations

This study was approved by the institutional review board for the protection of human subjects of Ono Academic College (201909ono).

## Supplementary Information

Supplementary Information.

## Data Availability

The datasets generated during and/or analyzed during the current study are available from the corresponding author on reasonable request.
