# Supplementary material for: The significance of right ear auditory processing to balance
Source: Sci Rep. 2022 Nov 17;12:19796. doi: 10.1038/s41598-022-24020-z (PMC9672090; doi:10.1038/s41598-022-24020-z)
Supplement: Supplementary file 1 — Supplementary Information. [file 41598_2022_24020_MOESM1_ESM.docx]

**SUPPLEMENTARY DATA**

Table S1. Regression results for simple mediation of right ear and left ear WIN 50% SNR on the association between age and balance

Table S2. Regression results for simple mediation of right ear and left ear PTA 1 on the association between age and balance.

Table S3. Regression results for simple mediation of right ear and left ear PTA 2 on the association between age and balance.

Table S4. Regression results for simple mediation of right ear and left ear 500Hz on the association between age and balance.

Table S5. Regression results for simple mediation of right ear and left ear 1000Hz on the association between age and balance.

Table S6. Regression results for simple mediation of right ear and left ear 2000Hz on the association between age and balance.

Table S7. Regression results for simple mediation of right ear and left ear 4000Hz on the association between age and balance

|  | *Right ear* | | | | | | *Left ear* | | | | | |
| --- | --- | --- | --- | --- | --- | --- | --- | --- | --- | --- | --- | --- |
| Variable | *β* | *SE* | *t* | *p* | *LLCI* | *ULCI* | *β* | *SE* | *t* | *p* | *LLCI* | *ULCI* |
| Direct and total effects | | | | | | | | | | | | |
| Balance regressed on age: (Path C) | .16 | .09 | 1.93 | .054 | -.003 | .332 | .19 | .09 | 2.23 | .027 | .022 | .358 |
| RE/LE WIN 50% SNR regressed on age: (Path A) | .26 | .06 | 4.69 | < .001 | .154 | .375 | .24 | .06 | 4.29 | < .001 | .131 | .354 |
| Balance regressed on RE/LE WIN 50% SNR, controlling for age: (Path B) | .25 | .09 | 2.97 | .003 | .086 | .421 | .17 | .09 | 2.01 | .046 | .003 | .339 |
| Balance regressed on age, controlling for RE/LE WIN 50% SNR: (Path C') | .16 | .09 | 1.93 | .054 | -.003 | .332 | .19 | .09 | 2.23 | .027 | .022 | .358 |

Table S1: Regression results for simple mediation of right ear and left ear WIN 50% SNR on the association between age and balance

*Note*. Unstandardized regression coefficients are reported. Bootstrap sample size = 5,000. LL = lower limit; CI = confidence interval; UL = upper limit;

RE = Right Ear; LE = Left Ear.

|  | *Right ear* | | | | | | *Left ear* | | | | | |
| --- | --- | --- | --- | --- | --- | --- | --- | --- | --- | --- | --- | --- |
| Variable | *β* | *SE* | *t* | *p* | *LLCI* | *ULCI* | *β* | *SE* | *t* | *p* | *LLCI* | *ULCI* |
| Direct and total effects | | | | | | | | | | | | |
| Balance regressed on age: (Path C) | .11 | .06 | 1.82 | .069 | -.009 | .224 | .12 | .06 | 2.09 | .038 | .007 | .241 |
| RE/LE PTA1 regressed on age: (Path A) | .27 | .06 | 4.89 | < .001 | .164 | .385 | .27 | .06 | 4.76 | < .001 | .157 | .379 |
| Balance regressed on RE/LE PTA1, controlling for age: (Path B) | .19 | .06 | 3.26 | .001 | .076 | .308 | .14 | .06 | 2.28 | .023 | .019 | .253 |
| Balance regressed on age, controlling for RE/LE PTA1: (Path C') | .11 | .06 | 1.82 | .069 | -.009 | .224 | .12 | .06 | 2.09 | .038 | .007 | .241 |

Table S2: Regression results for simple mediation of right ear and left ear PTA 1 on the association between age and balance

*Note*. Unstandardized regression coefficients are reported. Bootstrap sample size = 5,000. LL = lower limit; CI = confidence interval; UL = upper limit; RE = Right Ear; LE = Left Ear.

|  | *Right ear* | | | | | | *Left ear* | | | | | |
| --- | --- | --- | --- | --- | --- | --- | --- | --- | --- | --- | --- | --- |
| Variable | *β* | *SE* | *t* | *p* | *LLCI* | *ULCI* | *β* | *SE* | *t* | *p* | *LLCI* | *ULCI* |
| Direct and total effects | | | | | | | | | | | | |
| Balance regressed on age: (Path C) | .12 | .06 | 1.95 | .052 | -.001 | .240 | .13 | .06 | 2.10 | .037 | .008 | .247 |
| RE/LE PTA2 regressed on age: (Path A) | .35 | .06 | 6.42 | < .001 | .243 | .459 | .32 | .06 | 5.83 | < .001 | .213 | .431 |
| Balance regressed on RE/LE PTA2, controlling for age: (Path B) | .12 | .06 | 1.90 | .058 | -.004 | .237 | .10 | .06 | 1.68 | .093 | -.017 | .222 |
| Balance regressed on age, controlling for RE/LE PTA2: (Path C') | .12 | .06 | 1.95 | .052 | -.001 | .240 | .13 | .06 | 2.10 | .037 | .008 | .247 |

Table S3: Regression results for simple mediation of right ear and left ear PTA 2 on the association between age and balance

*Note*. Unstandardized regression coefficients are reported. Bootstrap sample size = 5,000. LL = lower limit; CI = confidence interval; UL = upper limit; RE = Right Ear; LE = Left Ear.

|  | *Right ear* | | | | | | *Left ear* | | | | | |
| --- | --- | --- | --- | --- | --- | --- | --- | --- | --- | --- | --- | --- |
| Variable | *β* | *SE* | *t* | *p* | *LLCI* | *ULCI* | *β* | *SE* | *t* | *p* | *LLCI* | *ULCI* |
| Direct and total effects | | | | | | | | | | | | |
| Balance regressed on age: (Path C) | .13 | .06 | 2.19 | .030 | .013 | .242 | .13 | .06 | 2.29 | .023 | .018 | .246 |
| RE/LE 500Hz regressed on age: (Path A) | .21 | .06 | 3.66 | < .001 | .097 | .321 | .17 | .06 | 2.95 | .003 | .057 | .283 |
| Balance regressed on RE/LE 500Hz, controlling for age: (Path B) | .16 | .06 | 2.70 | .007 | .043 | .272 | .17 | .06 | 2.87 | .004 | .052 | .280 |
| Balance regressed on age, controlling for RE/LE 500Hz: (Path C') | .13 | .058 | 2.19 | .030 | .013 | .242 | .13 | .06 | 2.29 | .023 | .018 | .246 |

Table S4: Regression results for simple mediation of right ear and left ear 500Hz on the association between age and balance

*Note*. Unstandardized regression coefficients are reported. Bootstrap sample size = 5,000. LL = lower limit; CI = confidence interval; UL = upper limit; RE = Right Ear; LE = Left Ear.

|  | *Right ear* | | | | | | *Left ear* | | | | | |
| --- | --- | --- | --- | --- | --- | --- | --- | --- | --- | --- | --- | --- |
| Variable | *β* | *SE* | *t* | *p* | *LLCI* | *ULCI* | *β* | *SE* | *t* | *p* | *LLCI* | *ULCI* |
| Direct and total effects | | | | | | | | | | | | |
| Balance regressed on age: (Path C) | .12 | .06 | 2.08 | .038 | .007 | .234 | .13 | .06 | 2.27 | .024 | .018 | .247 |
| RE/LE 1000Hz regressed on age: (Path A) | .20 | .06 | 3.43 | .001 | .084 | .309 | .19 | .06 | 3.34 | .001 | .079 | .304 |
| Balance regressed on RE/LE 1000Hz, controlling for age: (Path B) | .21 | .06 | 3.55 | < .001 | .092 | .319 | .15 | .06 | 2.53 | .012 | .033 | .262 |
| Balance regressed on age, controlling for RE/LE 1000Hz: (Path C') | .12 | .06 | 2.08 | .038 | .007 | .234 | .13 | .06 | 2.27 | .024 | .018 | .247 |

Table S5: Regression results for simple mediation of right ear and left ear 1000Hz on the association between age and balance

*Note*. Unstandardized regression coefficients are reported. Bootstrap sample size = 5,000. LL = lower limit; CI = confidence interval; UL = upper limit; RE = Right Ear; LE = Left Ear.

|  | *Right ear* | | | | | | *Left ear* | | | | | |
| --- | --- | --- | --- | --- | --- | --- | --- | --- | --- | --- | --- | --- |
| Variable | *β* | *SE* | *t* | *p* | *LLCI* | *ULCI* | *β* | *SE* | *t* | *p* | *LLCI* | *ULCI* |
| Direct and total effects | | | | | | | | | | | | |
| Balance regressed on age: (Path C) | .12 | .06 | 1.95 | .052 | -.001 | .236 | .13 | .06 | 2.14 | .033 | .011 | .246 |
| RE/LE 2000Hz regressed on age: (Path A) | .31 | .06 | 5.66 | < .001 | .205 | .423 | .28 | .06 | 4.94 | < .001 | .167 | .388 |
| Balance regressed on RE/LE 2000Hz, controlling for age: (Path B) | .14 | .06 | 2.27 | .024 | .018 | .255 | .12 | .06 | 1.96 | .051 | -.001 | .234 |
| Balance regressed on age, controlling for RE/LE 2000Hz: (Path C') | .12 | .06 | 1.95 | .052 | -.001 | .236 | .13 | .06 | 2.14 | .033 | .011 | .246 |

Table S6: regression results for simple mediation of right ear and left ear 2000Hz on the association between age and balance

*Note*. Unstandardized regression coefficients are reported. Bootstrap sample size = 5,000. LL = lower limit; CI = confidence interval; UL = upper limit; RE = Right Ear; LE = Left Ear.

|  | *Right ear* | | | | | | *Left ear* | | | | | |
| --- | --- | --- | --- | --- | --- | --- | --- | --- | --- | --- | --- | --- |
| Variable | *β* | *SE* | *t* | *p* | *LLCI* | *ULCI* | *β* | *SE* | *t* | *p* | *LLCI* | *ULCI* |
| Direct and total effects | | | | | | | | | | | | |
| Balance regressed on age: (Path C) | .16 | .06 | 2.55 | .011 | .036 | .279 | .16 | .06 | 2.55 | .011 | .036 | .279 |
| RE/LE 4000Hz regressed on age: (Path A) | .36 | .05 | 6.51 | < .001 | .248 | .463 | .35 | .06 | 6.48 | < .001 | .246 | .461 |
| Balance regressed on RE/LE 4000Hz, controlling for age: (Path B) | .01 | .06 | .14 | .892 | -.113 | .130 | .01 | .06 | .13 | .893 | -.113 | .130 |
| Balance regressed on age, controlling for RE/LE 4000Hz: (Path C') | .16 | .06 | 2.55 | .011 | .036 | .279 | .16 | .06 | 2.55 | .011 | .036 | .279 |

Table S7: Regression results for simple mediation of right ear and left ear 4000Hz on the association between age and balance

*Note*. Unstandardized regression coefficients are reported. Bootstrap sample size = 5,000. LL = lower limit; CI = confidence interval; UL = upper limit; RE = Right Ear; LE = Left Ear.
